# Psychometric properties of the Maladaptive Daydreaming Scale in a sample of Hungarian daydreaming-prone individuals

**DOI:** 10.1556/jba-9-853

**Published:** 2020-10-06

**Authors:** Alexandra Sándor, Ákos Münnich, Judit Molnár

**Affiliations:** 1 Department of Behavioural Sciences, Faculty of Medicine, University of Debrecen, Móricz Zsigmond krt. 22., H-4032, Debrecen, Hungary; 2 Doctoral School of Health Sciences, University of Debrecen, Debrecen, Hungary; 3 Institute of Psychology, Faculty of Humanities, University of Debrecen, Egyetem tér 1., H-4032, Debrecen, Hungary

**Keywords:** Maladaptive Daydreaming Scale, adaptation, absorption, childhood trauma, Hungarian sample

## Abstract

*Objectives*: The aim of the study is to adapt the Maladaptive Daydreaming Scale (MDS-16) to Hungarian, assess its psychometric properties, and establish its cut-off score. In addition, the relationship between maladaptive daydreaming and adverse childhood experiences was examined. *Method*: Study participants were recruited online via snowball sampling. Based on three inclusion criteria (self-identified MDer status; control over daydreaming; frequency of daydreaming) 160 out of 494 respondents were included in the study. *Results*: Our results confirm both the high reliability and convergent validity of the questionnaire. The cut-off score of 60 percentiles can reliably discriminate between excessive and normal daydreamers. The general applicability of the MDS-16-HU was tested and confirmed by the use of the Adverse Childhood Experience Questionnaire (ACE-10), a short, self-report questionnaire. Its results showed that certain types of childhood adversities increase the likelihood of maladaptive daydreaming. *Conclusions*: The instrument is a valid and reliable measure, therefore it can serve as a useful screening tool in clinical practice. In addition, our findings highlighted the role of childhood adversities in the aetiology of maladaptive daydreaming.

## Introduction

Daydreaming is a common mental activity during which attention drifts from monitoring the external environment and fulfilling the task in hand towards inspecting the inner world (Singer, 2014). Daydreaming and fantasizing are universal phenomena that do not have a negative impact on most people's lives. In fact, this imaginative activity serves adaptive functions: it enhances learning, planning, and thinking, promotes creativity, and facilitates problem solving (Killingsworth & Gilbert, 2010; Zedelius & Schooler, 2016).

The pathological form of daydreaming, accompanied by distress and impaired functioning, was first described in 2002 (Somer, 2002). According to the definition of Somer (2002, p. 197), “maladaptive daydreaming is extensive fantasy activity that replaces human interaction and/or interferes with academic, interpersonal, or vocational functioning.” The phenomenon was later defined as a clinical condition which causes a significant waste of time and a feeling of a loss of control, hinders everyday functioning and the building and maintaining of relationships, and hampers educational and vocational progress (Schimmenti, Somer, & Regis, 2019; Somer, Somer, & Jopp, 2016a). Although the problematic form of daydreaming was described more than 15 years ago, many concerns have remained regarding the aetiology, pathogenesis and treatment of the disorder.

The literature of maladaptive daydreaming indicates that the etiological conceptualization of the phenomenon is still inconsistent. Based on the reported characteristics, maladaptive daydreaming can be described as a multifaceted clinical phenomenon (Somer, 2018). The existing theories regarding the conceptualization are the following: learning theory; coping in response to childhood trauma; dissociative absorption; addiction and compulsion (Bigelsen, Lehrfeld, Jopp, & Somer, 2016; Bigelsen & Schupak, 2011; Pietkiewicz, Nęcki, Bańbura, & Tomalski, 2018; Somer, 2002, 2018; Somer & Herscu, 2017; Somer et al., 2016a; Somer, Somer, & Jopp, 2016b).

The seminal paper (Somer, 2002) revealed that the most important function of maladaptive daydreaming is to escape from reality and from painful experiences into a protective and comforting fantasy world. This study (Somer, 2002) suggested that maladaptive daydreaming might be explained as avoidant behaviour of real life threats which is rewarded and maintained by negative reinforcement (Mowrer, 1951).

Based on the same paper (Somer, 2002), there is another explanation of the development of maladaptive daydreaming, namely the trauma origin hypothesis. Excessive daydreaming might be a consequence of adverse childhood circumstances, and might function as a useful coping strategy to distance from these painful circumstances, as in the case of other dissociative reactions (Somer, 2002; Somer et al., 2016a). Later researchers reported that childhood traumatization is neither necessary nor sufficient for the emergence of this disorder (Biegelsen et al., 2016; Bigelsen & Schupak, 2011). However, in a recent study (Somer & Herscu, 2017), which explored a potential mediation model of daydreaming, researchers suggested that childhood trauma might be an independent risk factor for maladaptive daydreaming, but this relationship is mediated by absorption and fantasy addiction.

The first study on maladaptive daydreaming (Somer, 2002) also highlights similarities between dissociation and maladaptive daydreaming, suggesting that both of them could develop as a coping strategy against negative or adverse circumstances. Somer (2018), based on the results of previous research showing a strong significant correlation between maladaptive daydreaming and dissociative absorption (Somer, Lehrfeld, Bigelsen, & Jopp, 2016), suggests that maladaptive daydreaming might be placed at the pathological end of the absorption spectrum.

Further evidence (Bigelsen & Schupak, 2011) revealed that maladaptive daydreaming is so rewarding that it may become an addictive or compulsive behaviour. Excessive daydreamers experience many symptoms related to addictive or compulsive phenomena, such as intense yearning for daydreaming, impaired control over the activity, and withdrawal when they are unable to daydream as much as they would like to (e.g. anxiety, irritation, stomach ache). Results (Bigelsen & Schupak, 2011) highlight that although maladaptive daydreamers report the benefits of fantasy (e.g. happiness, calm), they experience intense distress as well, due to the amount of time diverted from real tasks and relationships, a feeling of shame about daydreaming and about their secretive behaviour, as well as the loss of control over the urge to daydream.

Many maladaptive daydreamers described an addictive vicious cycle; in cases of anxiety, distress and discomfort they tend to seek relief in their daydreams, but its compulsive, time consuming and shameful nature causes further distress (Somer et al., 2016b). Somer and Herscu (2017) in their article already identify the phenomenon as a behavioural addiction. Their mediation model suggests that addiction to fantasy and absorption are important central factors in the development of maladaptive daydreaming. A recent case study (Pietkiewicz et al., 2018) shows that maladaptive daydreaming is a coping strategy to avoid the experience of distress and pain, and to cope with traumatic experiences, negative emotions, social rejection and loneliness. This excessive form of daydreaming might share similar features with other addictive disorders such as Internet gaming disorder.

During the past two or three decades, the number of research studies dealing with problematic, excessive behaviours has significantly increased, a phenomenon which, however, might lead to an endless list of addictive behaviours and to the over-pathologization of everyday activities (Billieux, Schimmenti, Khazaal, Maurage & Heeren, 2015). According to a new approach to behavioural addiction, in order to avoid pathologizing common behaviours and leisure activities (such as video gaming, excessive exercising or dancing), and to increase the relevance and credibility of addictions, a clear theoretical and methodological framework is needed (Billieux et al., 2015; Kardefelt-Winther et al., 2017).

Based on a recent operational definition of behavioural addiction, several requirements are necessary to categorize a behaviour as an addictive disorder: a clear separation from normative behaviours (also from those which are characterized by high engagement or passion), evidence of serious functional impairment or psychological distress caused by the excessive behaviour, and the persistence of the behaviour over time. Furthermore, four exclusion criteria were added to the definition of behavioural addiction to avoid the false identification of excessive behaviours: (1) the behaviour can be explained by an underlying disorder; (2) the activity causing functional impairment is based on willful choice; (3) the behaviour is so intense that it reduces the amount of time and focus dedicated to other aspects of life, although it does not cause functional impairment or distress; (4) the activity is a result of a helpful or a maladaptive coping strategy (Kardefelt-Winther et al., 2017).

At the beginning of research into maladaptive daydreaming, an important starting point was to avoid the pathologization of a common mental activity. However, the findings of many studies provided evidence for the clear separation of maladaptive daydreaming from normal daydreaming as it impairs daily functioning and social relations and causes health-related dysfunctions (Somer et al., 2016).

Based on the operational definition of behavioural addiction, maladaptive daydreaming can be differentiated from adaptive and constructive daydreaming activities as this clinical syndrome causes a significant waste of time, intense yearning, shame and guilt, and also interferes with life functioning as it impairs the academic, professional and interpersonal aspects of life (Bigelsen et al., 2016; Bigelsen & Schupak, 2011; Somer, 2002). Furthermore, maladaptive daydreaming differs from normative daydreaming regarding the content, controllability, quantity, experience, and level of distress caused by this excessive activity (Bigelsen et al., 2016). Many maladaptive daydreamers reported an early onset of excessive daydreaming, as they discovered their vivid fantasizing capacity during their childhood. However, excessive daydreaming activity in many cases continued during adulthood, causing severe distress to the individuals (Somer et al., 2016a), which confirms the persistency criterion of behavioural addictions (Kardefelt-Winther et al., 2017). Regarding the four exclusion criteria, (1) associations between maladaptive daydreaming and other disorders (e.g. obsessive-compulsive disorder, dissociative disorders, attention-deficit/hyperactivity disorder) revealed similarities and differences in characteristics, confirming the clinical validity of maladaptive daydreaming (Somer et al., 2016), which “appears to be a behavioural addiction to absorptive fantasy” (Somer, Soffer-Dudek, Ross & Halpern, 2017, p. 186). The high comorbidity of maladaptive daydreaming with other disorders, according to Somer, Soffer-Dudek, Ross, and Halpern (2017), is not an invalidating factor, as many disorders of Diagnostic and Statistical Manual of Mental Disorders, Fifth Edition (DSM-5) show high rates of comorbidity as well, and even this finding confirmed that maladaptive daydreaming is a form of psychopathology. Based on their results, Somer et al., (2017) concluded that “maladaptive daydreaming cannot be better accounted for by any other existing DSM-5 disorder” (p. 529). Maladaptive daydreaming is a multifaceted phenomenon; however, it is still a challenge for researchers and clinicians to understand the essence of the disorder (Somer, 2018). (2) This excessive activity is characterized by loss of control, and often appears against the will of the individual (Bigelsen & Schupak, 2011; Somer et al., 2016a, 2016b). Many maladaptive daydreamers experience that this activity has an addictive quality and because of the impaired self-care and negative health outcomes (such as sleep disturbance), this form of daydreaming is not compatible with normal life (Somer et al., 2016a). (3) Maladaptive daydreamers reported that this activity causes significant waste of time and interferes with other aspects of life; moreover, it causes severe functional impairment and psychological distress (Bigelsen et al., 2016; Bigelsen & Schupak, 2011; Somer, 2002; Somer et al., 2016, 2016a, 2016b). (4) Although maladaptive daydreaming was first described as a strategy to cope with pain, negative emotions and adverse life experiences, and as a source of intimacy, loving, soothing experiences and emotions (Somer, 2002), research has revealed that the phenomenon is a complex psychological syndrome, which itself is a source of distress, isolation and maladaptation (Bigelsen & Schupak, 2011; Somer et al., 2016a). Recent studies have explored the idea that neither the immersive nor the maladaptive components of maladaptive daydreaming are effective emotion regulation strategies, and problematic daydreamers have poor and limited emotion regulation skills (Greene, West, & Somer, 2020; West & Somer, 2019).

According to Kardefelt-Winther and his colleagues (2017), in the case of “new” problematic behaviours, research should focus on their phenomenology and aetiology in order to provide valid and reliable evidence about their addictive nature and to avoid over-pathologizing and stigmatizing normative behaviours. This might be an important direction for maladaptive daydreaming research, as well.

Although the publication of the original paper received great attention from those affected by maladaptive daydreaming (Bershtling & Somer, 2018), who have created hundreds of web pages, forums and online communities in order to share information and find peers, the phenomenon has remained under-researched. At the time of writing this article, a total of 32 studies had been published in the international literature, while in Hungary only a few studies have attempted to examine the pathological form of daydreaming (Sándor & Molnár, 2018; Zsila, McCutcheon, & Demetrovics, 2018; Zsila, Urbán, McCutcheon, & Demetrovics, 2019).

The aim of the present study was to adapt the Maladaptive Daydreaming Scale (MDS-16, Somer et al., 2017), examine its psychometric properties, and determine its cut-off score in a Hungarian sample. After determining the cut-off score of the MDS-16-HU, we intended to test its applicability as well. We used the Hungarian version of the Adverse Childhood Experience Questionnaire (ACE-10), based on two considerations. Firstly, the ACE-10 questionnaire is a short, 10-item questionnaire that seemed to be a good choice to test the applicability of the Hungarian cut-off score of the MDS-16-HU. Secondly, as trauma origin is one of the possible etiological factors behind maladaptive daydreaming, comparing the results of the ACE-10 scores of maladaptive daydreamers and non-problematic daydreamers promised to provide some new information about this question as well.

## Method

### Sampling procedure

Online snowball sampling was carried out to recruit research participants, who could access the questionnaire package via an Internet link. The research call was advertised on the general platform of Facebook, as well as in specific Facebook communities dedicated to daydreaming and/or psychological topics. Furthermore, we invited members of a closed online group created by our research team in 2015. This group (Excessive daydreaming–Maladaptive daydreaming) was initiated to share information about the phenomenon of maladaptive daydreaming with the members of the group and to announce invitations to participate in subsequent research. Online data collection was justified by the fact that the target group of our research has hardly been accessible in any other way. Previous studies have shown that maladaptive daydreamers feel profound shame and are extremely embarrassed about their behaviour, as well as being strongly motivated to conceal their activity even from their closest relatives or friends, and are so worried about being possibly humiliated that they are reluctant to talk about their pathological fantasy activity even in a trusting therapeutic relationship (Bigelsen & Schupak, 2011; Schimmenti, Sideli, La Marca, Gori, & Terrone, 2019; Somer et al., 2016a).

The results of previous international research on maladaptive daydreaming (Bigelsen et al., 2016; Bigelsen & Schupak, 2011; Soffer-Dudek & Somer, 2018; Somer et al., 2016; West & Somer, 2019) also suggest that online assessment is adequate as it is based on a voluntary approach, ensures complete anonymity, and encourages open and truthful answers. The responses of 494 individuals (414 women and 80 men) were included in the study. Given the fact that there is no diagnostic tool available in Hungarian for the assessment of maladaptive daydreaming, we applied three criteria to identify excessively daydreaming individuals. One of our inclusion criteria was the self-identified MDer status, which had been applied by former international studies as the only screening criterion (Abu-Rayya, Somer, & Meari-Amir, 2019; Bigelsen et al., 2016; Bigelsen & Schupak, 2011; Jopp, Dupuis, Somer, Hagani, & Herscu, 2018; Schimmenti et al., 2019; Somer et al., 2016a, 2016b). In the questionnaire package we used the definition of maladaptive daydreaming based on the definition applied in several maladaptive daydreaming studies (Bigelsen et al., 2016, p. 257; Somer et al., 2016b, p. 563): “Do you consider the phenomenon of maladaptive daydreaming to be typical of you? These are fantastical mental images and narratives that are not currently part of your life. Excessive daydreaming might cause distress or impair everyday functioning.” Study subjects could consider whether maladaptive daydreaming was typical of them or not (i.e. could choose either a “yes” or “no” option). In our research, two additional criteria were added: the observed degree of control over daydreaming and the frequency of daydreaming. These two criteria accompanied the frequently used inclusion criteria (self-identification) for a more accurate and reliable identification of the study groups.

The degree of control over daydreaming could be defined by respondents on a scale of 0 (completely uncontrollable) to 10 (completely controllable). The feeling of loss of control over the fantasy activity is one of the key traits of maladaptive daydreaming; this can most effectively distinguish maladaptive from normal daydreaming. Maladaptive daydreamers are unable to resist yearning, will keep returning to their fantasy world again and again, and all the attempts made to suspend daydreaming will fail (Bigelsen & Schupak, 2011). The excessive frequency of daydreaming and its time-consuming nature is a further key factor of this phenomenon. Thus, in the case of the third inclusion criterion, respondents were provided five options to choose from (once a day, several times a day, once a week, several times a week, a few times a month).

The participants who simultaneously fulfilled all the three criteria described above, that is they identified themselves as daydreaming excessively, had a degree of observed control over daydreaming ranging from 0 to 5 (out of 10), and admitted daydreaming at least once a day or several times a day, were included in the study group of excessive daydreamers. However, those respondents who did not consider the definition of excessive daydreaming to be true for themselves, felt this activity highly controllable (ranging from 6 to 10), and said they only daydreamed once a week or a few times a month served as the comparison group. Based on the three criteria, a total of 70 respondents were included in the group of excessive daydreamers and 90 were included in the comparison group.

### Participants

The final study sample consisted of 160 participants (16.25% male; 83.75% female) based on the three a priori-defined criteria (self-identification, observed control over daydreaming, frequency of daydreaming), 70 of whom proved to be excessive daydreamers, and 90 of whom were normal daydreamers.

The youngest respondent was 18 and the oldest was 68 (M = 33.77; SD = 11.09). As regards marital status, those living in a relationship (64.38%) were over-represented among the respondents (partnership: 26.88%; registered partnership: 15.63%; marriage: 21.88%); at the time of the assessment 29.38% were single, 4.38% were divorced, and 1.88% were widows/widowers. Regarding education, more than half of our sample (63.13%) had a university degree (college: 15%; university: 39.38%; postgraduate studies: 8.75%), 31.88% had General Certificate of Secondary Education (GCSE-level qualifications), and those who had only completed their elementary school education accounted for 0.63% of the sample. The category of “other educational attainment” (4.38%) included respondents who had completed a vocational school (1.88%), technician school (1.25%) or received higher vocational training (1.25%). In terms of employment, nearly half of the respondents (46.25%) work, 23.75% of them work and study at the same time, 18.13% study, 5.63% are currently unemployed, and the category called “Other” (6.25%) includes pensioners (2.5%), working pensioners (0.63%), homemakers (1.25%), people on child care leave (0.63%) and those receiving child care benefit (1.25%). 20.63% of the respondents admitted that they had received or had been receiving psychiatric care, and 28.75% had taken part in psychotherapy. 63.13% had felt it necessary to turn to a psychiatrist or psychologist, and 21.88% had taken or were on some psychiatric medication. In addition, 26.25% of the respondents had a close family member who had already received psychiatric care.

### Measures

#### Demographic and basic clinical information

Participants were asked for basic demographic data, and also provided clinical information related to the need for requesting psychological assistance, their participation in previous or current psychiatric and/or psychological treatment, psychiatric medication, and the participation of their close family members in psychiatric care.

#### The Maladaptive Daydreaming Scale (MDS-16)

To discriminate between maladaptive daydreaming and normal daydreaming, we used the Maladaptive Daydreaming Scale (MDS-16, Somer et al., 2017). The self-report screening questionnaire contains 16 items, which the respondents are asked to answer on a scale ranging from 0 to 100%, with 10% intervals. The measure was translated into Hungarian by two independent translators, and then a third individual (an English to Hungarian translator specialized in Psychology) compared the two translations to the original scale and gave some proposals on modifications. After the three translators had reached a consensus, another English to Hungarian translator specialized in Psychology translated the scale back into the original language. The comparison of the back translated scale and the original questionnaire was made by the third person, who also submitted proposals on the design of the final format of the scale, which was discussed with the translators and the specialized translator as well.

#### The Tellegen Absorption Scale (TAS)

The convergent validity of the Hungarian version of the Maladaptive Daydreaming Scale (MDS-16-HU) was examined with the Tellegen Absorption Scale (TAS, Tellegen & Atkinson, 1974). The data in the literature data shows that maladaptive daydreaming and absorption are correlated (Bigelsen et al., 2016; Somer et al., 2016a; Somer & Herscu, 2017); evidence suggests that maladaptive daydreaming can be identified as a highly absorptive activity (Somer, 2018). Absorption is a disposition for having an experience of total attention resulting in a higher level of the sense of the attentional object, as well as an altered sense of reality and altered perception of the self (Tellegen & Atkinson, 1974). Although both phenomena facilitate the experience of intense and fanciful imagery with sensory and affective properties, absorption is a capacity which enables an intensified sense of positive and negative feelings, body sensations, events and recall of memories (Simor, Köteles, & Bódizs, 2011), while maladaptive daydreaming is an absorptive but addictive psychiatric disorder causing distress, withdrawal and shame (Pietkiewicz et al., 2018).

When filling in the 34-item survey, respondents could choose from two options (true–false). Researchers assessed the reliability of the TAS in a Hungarian sample of undergraduate students and their results suggested that the scale had good internal consistency (Cronbach *α* = 0.86) and that absorption was related to several personality dimensions and characteristics such as fantasy and emotional openness, dissociative experiences, and private self-consciousness (Simor et al., 2011).

#### The shortened version of the Adverse Childhood Experience Questionnaire (ACE-10)

Traumatic childhood experiences were assessed with the 10-item ACE score calculator (Adverse Childhood Experience Questionnaire–Finding Your ACE Score, Anda, Butchart, Felitti, & Brown, 2010; translated to Hungarian by Anikó Ujhelyiné Nagy and Ildikó Kuritárné Szabó in 2015), which assesses five experiences of abuse (emotional, physical and sexual abuse, and emotional and physical neglect), as well as five categories of household dysfunction [parental separation or divorce, domestic violence towards the mother or foster mother (witnessed violence), familial alcohol abuse or other psychoactive substance abuse, familial mental illness or suicide attempt, household incarceration].

#### The structured questionnaire of daydreaming experience

Based on the results of previous studies (Bigelsen, Lehrfeld, Jopp, & Somer, 2016; Bigelsen & Schupak, 2011), we compiled a 23-item questionnaire to reveal some specific features of daydreaming in detail. The structured questionnaire on daydreaming contained questions about the characteristics of daydreaming (frequency, duration, time of the day, location, body position, movements, listening to music, onset), as well as about the observed control over daydreaming; then the respondents had open questions to talk about the content and imaginary title of their daydreams, the feelings they experienced, the benefits and disadvantages of daydreaming, and what the daydreaming activity meant for them. We also asked them to describe the factors that trigger or maintain this mental activity, and to write about the conditions and factors that help them stop it; daydreamers also described how daydreaming enhanced or impaired their life. Qualitative content analysis will be applied to examine the answers.

### Data analytic strategy

Cronbach's alfa was applied to measure the reliability of the Hungarian version of the Maladaptive Daydreaming Scale. A previous study (Somer et al., 2016) had suggested that the items of the MDS-14 are thematically and substantively highly related to each other, and they found strong evidence for using a single composite score. In line with Somer's indication (Somer et al., 2016) we used the average total MDS score for the calculations.

We assessed the reliability of the TAS, and the Pearson correlation coefficient was calculated in order to measure the interrelationship between maladaptive daydreaming and absorption.

When attempting to determine the cut-off score, we used cross tables to examine the allocation of respondents at different percentiles, and used a chi-square statistic to measure the correspondence. The ultimate cut-off score is defined by the score at which the chi-square value is at a maximum.

Cross tables were applied in order to investigate the relationship between the MDS-16-HU and the individual items of the ACE-10; then we performed Fisher's exact test to assess the significance of the correlation.

### Ethics

The research was conducted in line with the Helsinki Declaration and approved by the Regional and Institutional Research Ethics Committee at the Clinical Center of the University of Debrecen. The online questionnaire package could only be accessed after the participants read the research information and gave their informed consent to participation in the study.

## Results

### The reliability and convergent validity of the Maladaptive Daydreaming Scale

The value of the Cronbach's *α* coefficient used to examine the internal consistency of the scale (Cronbach *α* = 0.957) indicates that the questionnaire is highly reliable.

The reliability of the TAS was very high (Cronbach *α* = 0.847). We found a moderate, significant correlation between the overall score of the MDS-16-HU and the overall score of the TAS (Pearson correlation coefficient value: *r* (160) = 0.448 (*p* < 0.001)), which confirmed our hypothesis that the two scales measure similar yet not identical phenomena, which supports the convergent validity of the MDS-16-HU.

### Determining the cut-off score of the MDS-16-HU

We used cross-tables to compare the a priori allocation of the respondents based on the three inclusion criteria (self-identification, control over daydreaming and frequency of daydreaming) and categorizations of the MDS cut-off scores. Table 1 shows the resulting cross-table.

**Table 1.** Comparison between the allocation based on the cut-off score (60 percentiles) and the a priori categorization

	Number of maladaptive daydreamers based on three criteria	Number of normal daydreamers based on three criteria	Overall
Number of maladaptive daydreamers based on the cut-off score	61	2	63
Number of normal daydreamers based on the cut-off score	9	88	97
Overall	70	90	160

Then we identified an optimal cut-off score (percentile), in the position where the maximum value of the chi-squared could be obtained. Tables 2 and 3 illustrate the usage of chi-square statistic for determining the cut-off score. The maximum chi-square value was obtained at 60 percentiles (a cut-off score of 35), as it was 118.95.

**Table 2.** Calculation of the MDS cut-off scores from 10 to 90 percentiles in increments of 5 percentiles

Percentile	Chi-square value	Cut-off point
10	14.794	3.75
15	24.15	6.875
20	32.336	8.125
25	41.482	10
30	54.935	11.25
35	68.867	13.125
40	81.429	15.625
45	95.456	18.75
50	106.906	21.25
55	115.158	25
60	118.953	35
65	103.848	38.75
70	85.563	45
75	64.075	51.25
80	49.435	57.5
85	34.536	61.875
90	21.281	68.125

**Table 3.** Calculation of the MDS cut-off scores from 55 to 65 percentiles in increments of 1 percentile

Percentile	Chi-square value	Cut-off point
55	115.158	25
56	125.487	28.75
57	121.911	29.375
58	122.037	30.625
59	118.602	34.375
60	118.953	35
61	115.647	35.625
62	109.233	36.875
63	106.122	38.125
64	103.848	38.75
65	101.896	40

### The findings of the Adverse Childhood Experience Questionnaire (ACE-10) questionnaire

We established the two study groups, maladaptive and normal daydreamers' groups, on the basis of the Hungarian cut-off score, i.e. 60 percentiles.

**Fig. 1.**
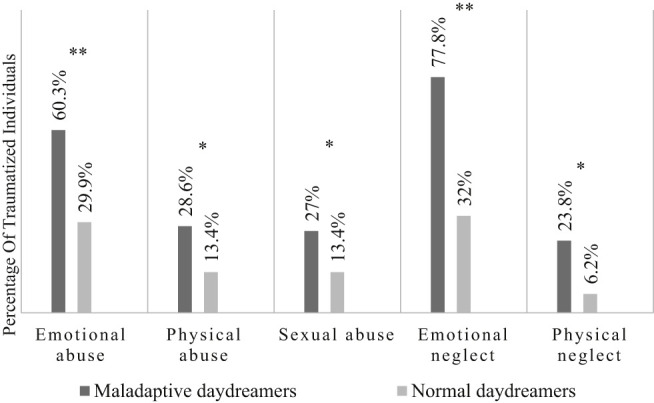
Comparing the prevalences of childhood abuse and neglect in the two groups. Fig. 1 Investigation of the correlation between the MDS-16-HU and the first five items of the ACE-10. Fisher's exact test was performed to assess the significance of the correlation. *P < 0.05; **P < 0.001

Based on the results of the cross tables and Fisher’s exact test, we found the first five trauma types to be risk factors for maladaptive daydreaming since emotional, physical and sexual abuse, as well as emotional and physical neglect experienced in childhood, significantly increased the likelihood of developing maladaptive daydreaming rather than normal daydreaming. The correlation is shown in Fig. 1.

Sixtyseven respondents in our sample (41.88%) had been abused emotionally in their childhood, 38 of whom (56.72%) showed a predisposition to excessive daydreaming. 31 respondents in our sample (19.38%) had suffered physical abuse; 18 of them (58.06%) were found to be maladaptive daydreamers. 30 respondents (18.75%) had been abused sexually, and 17 of them (56.67%) became excessive daydreamers. 80 respondents, i.e. half of the participants, had been neglected in their childhood, 49 of whom (61.25%) exhibited a predisposition to maladaptive behaviour; 21 respondents (13.13%) had been subject to physical neglect and 15 of them (71.43%) were found to be maladaptive daydreamers.

**Fig. 2.**
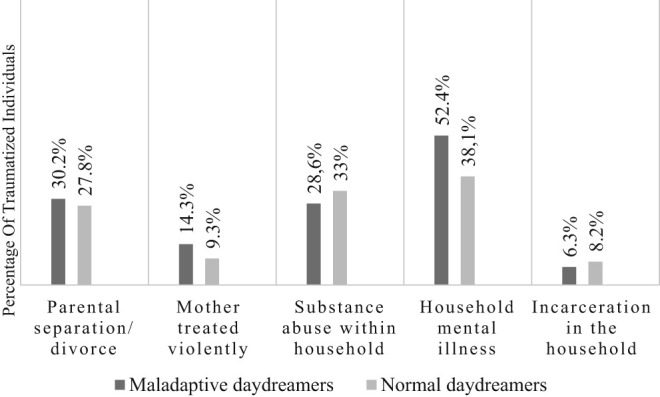
Comparing the prevalences of household dysfunction in the two groups. Fig. 2 Investigation of the correlation between the MDS-16-HU and the last five items of the ACE-10. Fisher's exact test was performed to assess the significance of the correlation

However, we did not find a significant correlation between the familial dysfunctions experienced in childhood and maladaptive daydreaming, thus they did not prove to be predisposing factors for problematic daydreaming. There is one type of trauma worth highlighting: the presence of a family member who suffers from mental disorder or has attempted to commit suicide. This circumstance may increase the risk of excessive daydreaming, though according to the result of the Fisher's exact test the correlation is not significant. The results are shown in Fig. 2.

As regards household dysfunction, parental separation or divorce had been experienced by 46 respondents in our sample (28.75%), 19 of whom (41.3%) were found to be maladaptive daydreamers. Eighteen respondents (11.25%) had witnessed violence against their mother or foster mother, and 9 of them (50%) later became excessive daydreamers. Fifty participants (31.25 %) had a family member who consumed alcohol or some other substance excessively, 18 of whom (36%) exhibited a tendency towards excessive daydreaming. A total of 70 respondents (43.75%) had some family member who was mentally ill or had attempted suicide, and 33 of these respondents (47.14%) tended to be maladaptive daydreamers. Incarceration of some family member was true for 12 participants (7.5%), 4 of whom (33.33%) proved to be maladaptive daydreamers.

## Discussion

The 16-item version of the Maladaptive Daydreaming Scale (MDS-16) was administered in the present study. We translated the scale into Hungarian, and our results confirmed the high reliability and convergent validity of the MDS-16-HU. The validity scores of the measure showed that the tool is appropriate for the identification of excessive daydreamers. Those with a high score on the scale exhibit a tendency towards maladaptive daydreaming; however, as the measure should only be considered a screening survey rather than a diagnostic tool, it is not sufficient to establish a diagnosis. Our results show that the cut-off score of 35 on the scale (60 percentiles) can effectively discriminate between excessive and normal daydreamers. Allocation using this cut-off value and categorization based on three a priori-defined criteria (self-identified MDer status; control over daydreaming; frequency of daydreaming) overlap to a great extent (93.1%).

The applicability of the cut-off score established in the Hungarian sample was further tested by applying the ACE-10. The reason for our choice was that the trauma origin hypothesis of maladaptive daydreaming still remains to be answered. Previous results suggest that maladaptive daydreaming may be related to childhood trauma (Bigelsen et al., 2016; Bigelsen & Schupak, 2011; Somer, 2002; Somer et al., 2016b; Somer & Herscu, 2017). These studies, however, have not considered an examination of the connection between maladaptive daydreaming and the specific traumatic experiences.

The consistent results of the ACE-10 – significant correlation between Maladaptive Daydreaming and the five types of childhood abuse and neglect, whereas there is no significant connection between maladaptive daydreaming and the five types of household dysfunction – confirmed the reliable and valid applicability of the MDS-16-HU, which seemed to differentiate well between maladaptive and normal daydreamers. Our results revealed that traumatic childhood experiences such as emotional and physical neglect, and emotional, physical and sexual abuse significantly increase the probability of maladaptive daydreaming, whereas familial dysfunctions did not prove to be predisposing factors. We found childhood emotional neglect and emotional abuse to be the most relevant risk factors for developing maladaptive daydreaming. Childhood emotional abuse and neglect are forms of abuse and interactions that do not require physical contact with the child, yet, they cause immense harm in all fields of early development and functioning (Glaser, 2002). Emotional abuse can cause damage to children's emotional and physical health, as well as their physical, mental, social, moral and spiritual development (Butchart, Harvey, Mian, & Fürniss, 2006). In childhood, for those having a particular fantasizing capacity, immersive absorption in a self-created alternate world functions as a safe place or refuge and daydreaming activity enables children to escape from unacceptable harmful, painful interactions and experiences such as loneliness, isolation, and neglect (Somer, 2019). Our research reveals that one of the consequences of the abuse of children may be the creation of a compensating inner fantasy world to cope with reality, but it may become excessive and maladaptive, and cause insatiable yearning and loss of control (Somer et al., 2016a).

### Limitations

One limitation of the study is that female respondents and those with tertiary educational attainment were over-represented in our sample. Our study was based on online sampling since this was the method that could make it easier for participants to answer honestly and to completely avoid the occurrence of stigmatization. It may be a drawback of Internet based data collection that it was mostly the younger and middle-aged generation with good computer skills and Internet access that responded to our call. Future studies should expand the range of the participants involved in research by including more male subjects in the sample as well as more participants with primary or secondary educational attainment. All the questionnaires we employed were self-report surveys, thus bias may have occurred in the answers.

We aimed to provide a preliminary interpretation of the findings of the ACE-10. However, we believe that further research would be needed for a more detailed and more in-depth examination of the trauma origin of maladaptive daydreaming.

## Conclusions

This study proved the reliability and convergent validity of the MDS-16-HU. The cut-off score of 60 percentiles can effectively discriminate between excessive and normal daydreamers. The general applicability of the MDS-16-HU was also tested and confirmed by the use of the ACE-10, a short and self-report questionnaire, in a sample of 160 participants. Our results demonstrated the potential role of childhood trauma in the aetiology of maladaptive daydreaming and its maintenance. Our findings suggest that the assessment of adverse childhood experiences, in particular emotional neglect and abuse, may have an essential role regarding the psychotherapy of maladaptive daydreamers. Future studies should focus on traumatic childhood experiences as potentially important segments of the aetiology of maladaptive daydreaming, and reveal which specific trauma types could function as potential risk factors for developing problematic daydreaming.

We plan to carry out the qualitative analysis of our structured questionnaire at a later stage of the research. We believe that the study findings may contribute to further broadening knowledge of maladaptive daydreaming and also to assisting in the adequate screening and identification of this phenomenon.

## Funding sources

This research was supported in part by EFOP-3.6.3-VEKOP-16-2017-00009 co-financed by EU and the European Social Found.

## Author's contribution

All authors made substantial contribution to study concept and design, as well as to data analysis and interpretation. In details: **AS:** Investigation, Obtained funding, Data Curation, Writing-Original Draft; **ÁM:** Statistical analysis, Writing-Review and Editing; **JM:** Concept and design, Study supervision, Writing-Review and Editing. All authors have full access to all data in the study and take responsibility for the integrity of the data and the accuracy of the data analysis.

## Conflict of interest

The authors declare that there is no conflict of interest.

## Acknowledgements

We would like to express our gratitude to Professor Eli Somer, PhD, for his support and valuable suggestions regarding the research process.

## References

[B1] Abu-Rayya, H. M., Somer, E., & Meari-Amir, S. (2019). The psychometric properties of the Arabic 16-item Maladaptive Daydreaming Scale (MDS-16-AR) in a multicountry Arab sample. *Psychology of Consciousness: Theory, Research, and Practice*, 6(2), 171–183. 10.1037/cns0000183.

[B2] Anda, R. F., Butchart, A., Felitti, V. J., & Brown, D. W. (2010). Building a framework for global surveillance of the public health implications of adverse childhood experiences. *American Journal of Preventive Medicine*, 39, 93–98. 10.1016/j.amepre.2010.03.015. 20547282

[B3] Bershtling, O., & Somer, E. (2018). The micro-politics of a new mental condition: Legitimization in maladaptive daydreamers’ discourse. *Qualitative Report*, 23(8), 1983–2002.

[B4] Bigelsen, J., Lehrfeld, J. M., Jopp, D. S., & Somer, E. (2016). Maladaptive daydreaming: Evidence for an under-researched mental health disorder. *Consciousness and Cognition*, 42, 254–266. 10.1016/j.concog.2016.03.017. 27082138

[B5] Bigelsen, J., & Schupak, C. (2011). Compulsive fantasy: Proposed evidence of an under-reported syndrome through a systematic study of 90 self-identified non-normative fantasizers. *Consciousness and Cognition*, 20, 1634–1648. 10.1016/j.concog.2011.08.013. 21959201

[B6] Billieux, J., Schimmenti, A., Khazaal, Y., Maurage, P., & Heeren, A. (2015). Are we overpathologizing everyday life? A tenable blueprint for behavioral addiction research. *Journal of Behavioral Addictions*, 4(3), 119–123. 10.1556/2006.4.2015.009. 26014667 PMC4627665

[B7] Butchart, A., Harvey, A. P., Mian, M., & Fürniss, T. (2006). Preventing child maltreatment: A guide to taking action and generating evidence. Geneva: World Health Organization and International Society for Prevention of Child Abuse and Neglect. Retrieved from https://apps.who.int/iris/bitstream/handle/10665/43499/9241594365_eng.pdf?sequence=1&isAllowed=y.

[B8] Glaser, D. (2002). Emotional abuse and neglect (psychological maltreatment): A conceptual framework. *Child Abuse & Neglect*, 26, 697–714. 10.1016/s0145-2134(02)00342-3. 12201163

[B9] Greene, T., West, M., & Somer, E. (2020). Maladaptive daydreaming and emotional regulation difficulties: A network analysis. *Psychiatry Research*, 285, 112799. 10.1016/j.psychres.2020.112799. 32006907

[B10] Jopp, D. S., Dupuis, M., Somer, E., Hagani, N., & Herscu, O. (2018). Validation of the Hebrew version of the maladaptive daydreaming scale (MDS-H): Evidence for the generalizable measure of pathological daydreaming. *Psychology of Consciousness: Theory, Research, and Practice*, 6(3), 242–261. 10.1037/cns0000162.

[B11] Kardefelt-Winther, D., Heeren, A., Schimmenti, A., van Rooij, A., Maurage, P., Carras, M., (2017). How can we conceptualize behavioural addiction without pathologizing common behaviours? *Addiction*, 112(10), 1709–1715. 10.1111/add.13763. 28198052 PMC5557689

[B12] Killingsworth, M. A., & Gilbert, D. T. (2010). A wandering mind is an unhappy mind. *Science*, 330(6006), 932. 10.1126/science.1192439. 21071660

[B13] Mowrer, O. H. (1951). Two-factor learning theory: Summary and comment. *Psychological Review*, 58(5), 350–354. 10.1037/h0058956. 14883248

[B14] Pietkiewicz, I. J., Nęcki, Sz., Bańbura, A., & Tomalski, R. (2018). Maladaptive daydreaming as a new form of behavioral addiction. *Journal of Behavioral Addictions*, 7(3), 838–843. 10.1556/2006.7.2018.95. 30238787 PMC6426361

[B15] Sándor A., & Molnár J. (2018). Maladaptív álmodozás. In Sz. I. Kuritárné, J. Molnár, & A. Nagy, (Eds.), *Trauma-eredetű disszociáció: Elmélet és terápia* (pp. 285–302). Budapest: Oriold és Társai Kiadó.

[B16] Schimmenti, A., Sideli, L., La Marca, L., Gori, A., & Terrone, G. (2019). Reliability, validity, and factor structure of the maladaptive daydreaming scale (MDS–16) in an Italian sample. *Journal of Personality Assessment*, 102(5), 689–701. 10.1080/00223891.2019.1594240. 31012744

[B17] Schimmenti, A., Somer, E. & Regis, M. (2019). Maladaptive daydreaming: Towards nosological definition. *Annales Medico-Psychologiques*, 177, 865–874. 10.1016/j.amp.2019.08.014.

[B18] Simor P., Köteles F., & Bódizs R. (2011). Elmerülés az élményben: A Tellegen-féle Abszorpció Skála vizsgálata egyetemista mintán. *Mentálhigiéné és Pszichoszomatika*, 12(2), 101–123. 10.1556/Mental.12.2011.2.1.

[B19] Singer, J. L. (2014). Daydreaming and fantasy. London and New York: Routledge.

[B20] Soffer-Dudek, N., & Somer, E. (2018). Trapped in a daydream: Daily elevations in maladaptive daydreaming are associated with daily psychopathological symptoms. *Frontiers in Psychiatry*, 9(194), 1–14. 10.3389/fpsyt.2018.00194. 29867613 PMC5962718

[B21] Somer, E. (2002). Maladaptive daydreaming: A qualitative inquiry. *Journal of Contemporary Psychotherapy*, 32(2/3), 197–212. 10.1023/A:1020597026919.

[B22] Somer, E. (2018). Maladaptive daydreaming: Ontological analysis, treatment rationale; a pilot case report. *Frontiers in the Psychotherapy of Trauma and Dissociation**,* 1(2), 1–22. 10.XXXX/ftpd.2017.0006.

[B23] Somer, E. (2019). On dissociative identity disorder and maladaptive daydreaming. *Frontiers in the Psychotherapy of Trauma and Dissociation*, 3(1), 14–18. 10.XXXX/ftpd.2019.0020.

[B24] Somer, E., & Herscu, O. (2017). Childhood trauma, social anxiety, absorption and fantasy dependence: Two potential mediated pathways to maladaptive daydreaming. *Journal of Addictive Behaviors, Therapy & Rehabilitation*, 6(3), 1–5. 10.4172/2324-9005.1000170.

[B25] Somer, E., Lehrfeld, J., Bigelsen, J., & Jopp, D. S. (2016). Development and validation of the maladaptive daydreaming scale (MDS). *Consciousness and Cognition*, 39, 77–91. 10.1016/j.concog.2015.12.001. 26707384

[B26] Somer, E., Soffer-Dudek, N., Ross, C. A., & Halpern, N. (2017). Maladaptive daydreaming: Proposed diagnostic criteria and their assessment with a structured clinical interview. *Psychology of Consciousness: Theory, Research, and Practice*, 4(2), 176–189. 10.1037/cns0000114.

[B27] Somer, E., Somer, L., & Jopp, D. S. (2016a). Childhood antecedents and maintaining factors in maladaptive daydreaming. *The Journal of Nervous and Mental Disease*, 204(06), 474–478. 10.1097/NMD.0000000000000507. 27002749

[B28] Somer, E., Somer, L., & Jopp, D. S. (2016b). Parallel lives: A phenomenological study of the lived experience of maladaptive daydreaming. *Journal of Trauma & Dissociation*, 17(5), 561–576. 10.1080/15299732.2016.1160463. 26943233

[B29] Tellegen, A., & Atkinson, G. (1974). Openness to absorbing and self-altering experiences (‘absorption’), a trait related to hypnotic susceptibility. *Journal of Abnormal Psychology*, 83, 268–277. 10.1037/h0036681. 4844914

[B30] West, M. J., & Somer, E. (2019). Empathy, emotion regulation, and creativity in immersive daydreaming. *Imagination, Cognition and Personality: Consciousness in Theory, Research, and Clinical Practice*, 39(4), 358–373. 10.1177/0276236619864277.

[B31] Zedelius, C. M., & Schooler, J. W. (2016). The richness of inner experience: Relating styles of daydreaming to creative processes. *Frontiers in Psychology*, 6, 2063. 10.3389/fpsyg.2015.02063. 26869943 PMC4735674

[B32] Zsila, Á., McCutcheon, L. E., & Demetrovics, Zs. (2018). The association of celebrity worship with problematic Internet use, maladaptive daydreaming, and desire for fame. *Journal of Behavioral Addictions*, 7(3), 654–664. 10.1556/2006.7.2018.76. 30221539 PMC6426373

[B33] Zsila, Á., Urbán, R., McCutheon, L.E., & Demetrovics, Zs. (2019). A path analytic review of the association between psychiatric symptoms and celebrity worship: The mediating role of maladaptive daydreaming and desire for fame. *Personality and Individual Differences*, 151. 10.1016/j.paid.2019.109511.

